# Oral Treatment With Ileal Spores Triggers Immunometabolic Shifts in Chicken Gut

**DOI:** 10.3389/fvets.2020.00629

**Published:** 2020-09-08

**Authors:** Graham A. J. Redweik, Michael H. Kogut, Ryan J. Arsenault, Melha Mellata

**Affiliations:** ^1^Department of Food Science and Human Nutrition, Iowa State University, Ames, IA, United States; ^2^Interdepartmental Microbiology Graduate Program, Iowa State University, Ames, IA, United States; ^3^Southern Plains Agricultural Research Center, USDA-ARS, College Station, TX, United States; ^4^Department of Animal and Food Sciences, University of Delaware, Newark, DE, United States

**Keywords:** SFB, *Salmonella*, IgA, gut permeability, kinome, T_H_17, spores

## Abstract

The animal gut is a major site affecting productivity via its role in mediating functions like food conversion and pathogen colonization. Live microorganisms like probiotics are widely used to improve poultry productivity. However, given that chicks receive their microbiota from the environment at-hatch, a bacterial treatment that can stimulate gut immune maturation in early life can benefit animal health. Thus, our lab has begun investigating alternative means to improve poultry health via single inoculation with microbial spores. In this study, we orally-inoculated day-old chicks with ileal scrapings (ISs) enriched for spores via chloroform treatment (SPORE) or non-treated (CON). At 3, 7, and 14 days post-inoculation (dpi), gut permeability was measured via FITC-dextran assay in serum. Additionally, small intestinal scrapings (SISs) were tested for *in vitro Salmonella* killing and total IgA. Lastly, distal ileum was either fixed or flash-frozen for microscopy or kinome peptide array, respectively. Using bacterial 16S rRNA gene sequencing, SPORE and CON inocula were highly-similar in bacterial composition. However, spores were detected in SPORE but not in CON inoculum. Segmented filamentous bacteria (SFB) filaments were observed in the distal ileum in SPORE birds as early as 3 dpi and all birds at 7 and 14 dpi. Additionally, SFB were detected via PCR in the ceca, colonizing all SPORE birds at 3 dpi. At 3 dpi, SPORE birds exhibited lower gut permeability vs. CON. In SPORE birds, SISs induced greater *Salmonella* growth *in vitro* at 3 dpi yet significantly-reduced *Salmonella* load at 7 and 14 dpi compared to CON in an IgA-independent manner. SPORE distal ileal tissue exhibited unique upregulation of several immunometabolic processes vs. CON birds, including innate (Toll-like receptor, JAK-STAT) and adaptive (T/B cell receptor, T_H_17 differentiation) immune pathways, PI3K/Akt signaling, mTOR signaling, and insulin-related pathways. Collectively, these data suggest oral inoculation with ileal spores generally-improved gut health.

**Importance:** We report that ileal, spore-forming commensal microbes have potent effects on ileum immunometabolism. Additionally, we identify a functional ileal phenotype in spore-treated chickens, which matched several of the observed immunometabolic changes and was associated with SFB colonization in the ileum.

## Introduction

The intestinal tract is considered the central site for optimizing the health and production of food animals (1). In chickens, this complex tissue system must absorb nutrients to energize functions like growth and egg production while simultaneously serving as a barrier to pathogenic bacteria like *Salmonella* (2). Thus, interventions at the gastrointestinal tract are effective options to maximize health and production potential. Supplements like probiotics are commonly given to poultry to further maximize feed efficiency and pathogen resistance. Current probiotics require continuous addition in feed due to their inability to persist for long periods (3). Some taxa like *Lactobacillus* have been found to serve as reservoirs for antibiotic resistance (4). This need to continually supplement probiotics in the feed as well as label inaccuracies like viability increases production costs for poultry producers (5, 6). Thus, a novel probiotic that can be deliverable in a viable state and confer benefits after only a single dose is needed and would be immensely beneficial for poultry farmers. Spore-forming bacteria like *Bacillus* have become popular probiotics given their ability to form durable endospores [reviewed in (7)]. Spore-forming members of the ileal microbiota like segmented filamentous bacteria (SFB) have been experimentally-demonstrated to have drastic yet positive impacts on mammalian gut immunity (8). Thus, we hypothesized that spores recovered from the ileum might have significant effects on the maturation of the chicken immune system.

Most immune-signaling pathways and critical metabolic processes are regulated by protein kinases. These enzymes catalyze the post-translation modification process of proteins known as phosphorylation. Kinomics is the global study of kinases and kinase signaling, as phosphorylation plays a crucial role in mediating most cellular signaling processes. Regulation of protein function through phosphorylation is observed in cellular processes that include metabolism, apoptosis, and signal transduction (9–11). The reversible nature of phosphorylation makes this biochemical event a critical feature and an effective mechanism for regulating protein behavior (10). The development of the chicken-specific kinome peptide array has provided an invaluable tool for exploring the gut health phenotype through phosphorylation-mediated signaling (12). In this study, we tested the probiotic potential of ileal spores in young chicks using multiple approaches, including assays for gut health, microscopy, and immunometabolic kinome profiling.

## Materials and Methods

### Ethics Statement

Animal experiments were approved by Iowa State University Institutional Animal Care and Use Committee, Log #s 18-386 and 19-072. Animal enrichments were added to open floor pens to minimize stress during experimental procedures. Euthanasia techniques (CO_2_ asphyxiation) followed the American Veterinary Medical Association Guidelines (2013).

### Inoculum Preparation

Methods were based on previous experiments to enrich for ileal spores in mice (13). Briefly, scrapings from the distal ileum and ileo-ceco-colic junction of two-week-old commercial layer pullets (Clearfield, IA; *n* = 10) were pooled and resuspended in PBS (3 mM EDTA). These commercial pullets were fed a corn-based SBM formula supplemented with wheat middling. Pooled scrapings were then treated (SPORE) or untreated (CON) with chloroform (3% total solution). Tubes were gently inverted and placed on ice for 3 h until returning to Iowa State University, where tubes were then incubated for 30 min at 37°C. Inocula were then injected with CO_2_ to remove chloroform from SPORE inoculum. After a subsequent incubation for 10 min at room temperature (RT), the top layer was transferred to fresh microcentrifuge tubes and centrifuged at 10,000 × g for 10 min. The supernatant was discarded and the pellet was resuspended in a peptone-glycerol solution, evenly pooled between groups, purified through a 5 μm filter, and stored at −80°C for 3 months prior to animal-inoculation. The entire processes for both inocula were done under aerobic conditions.

### 16S Sequencing and Analysis

Total DNA was isolated via bead-beating from a single SPORE and CON inocula replicate (given inocula were pooled prior to storage) using the DNeasy PowerSoil Kit (Qiagen). Notably, this kit lacks enzymatic digestion steps, although no differences in community composition were observed when compared to other commonly-used DNA extraction kits (14). Extracted DNAs were assessed for quality using a NanoDrop 2000 spectrophotometer (260 to 280 nm ratios), and concentrations were determined using a Qubit fluorometer via dsDNA broad range kit (ThermoFisher Scientific). Sample DNA concentration was then adjusted to 50 ng/μl in nuclease-free water, and library-prepared via MiSeq and HiSeq2500 kit (Illumina) following all manufacturer's instructions with 151 × 151 paired-end MiSeq sequencing (Illumina). DNAs were then sequenced via Illumina MiSeq (v3) at the Iowa State DNA facility. Using QIIME2 (version 2019.10) for 16S rRNA gene analysis, sequences were demultiplexed via QIIME2 demux emp-paired function and denoised via QIIME2 plugin DADA2. The number of good quality reads for taxonomic assignment ranged from 36,954 to 80,793 reads. SILVA database at the 99% operational taxonomic units (OTUs) spanning the V4 and V5 regions (515F, GTGYCAGCMGCCGCGGTAA; 926R, CCGYCAATTYMTTTRAGTTT) was used to classify each of the reads using QIIME2's feature-classifier function. The raw sequence reads is available in the NCBI Sequence Read Archive (SRA) repository with accession BioProject ID PRJNA637043.

### Spore Imaging

For negative-staining of spores, 2 μl of each intestinal scraping sample was applied to a carbon film copper grid and incubated for 30 s at RT. The supernatant was briefly wicked, and 2 μl of aqueous 2% uranyl acetate (UA) was applied to the grid and allowed to sit for 30 s. The UA was wicked, and the resulting thin film was allowed to dry. Images were taken using a JEOL JSM 2,100 scanning transmission electron microscope at 200 kV (jeol.com) with a Gatan One View camera (gatan.com).

### Chicken Treatment

One-day-old male and female specific pathogen-free White Leghorns (VALO, Adel, IA) were randomly placed into 2 pens (*n* = 21 birds/pen) in the same room. Immediately after placement, birds were orally inoculated with sodium bicarbonate and 50 μl inoculum (chloroform-treated, SPORE; non-chloroform-treated, CON). SPORE inoculum contained ~150 spores per 50 ul aliquot. Food and water were provided 30 min after treatment. Following inoculation, body weights were collected at days 1 (prior to inoculation) and 11.

### PCR

Intestinal scraping and ceca content samples were separately-homogenized for 20 min via bead-containing tubes. DNAs were then extracted via DNeasy Powersoil Kit, and DNA concentrations were evaluated via NanoDrop 2000. SFB-specific PCR primers (15) were as follows: forward primer, AGGAGGAGTCTGCGGCACATTAGC; reverse primer, TCCCCACTGCTGCCTCCCGTAG. All reactions used 50 ng DNA template and 5 μM each primer. An initial denaturing step was set at 95°C for 15 min, followed by 32 cycles of 95°C (30 s), 59°C (30 s), and 72°C (30 s) as previously described (15).

### Gut Permeability

At days 3, 7, and 14 post-inoculation, birds (*n* = 7 per time point) were orally inoculated with fluorescein isothiocyanate dextran (FITC-d, MW 3–5 kDa; Sigma Aldrich; 8.32 mg/kg chicken) 2 h prior to sacrifice. Serum from all FITC-d-inoculated birds was collected via wing vein, subsequently-centrifuged, and kept at 4°C until ready to aliquot onto 96 well-plates. A standard curve using serum from naïve birds serially-diluted for specific, added FITC-d concentrations (6,400 to 0 ng/ml) was developed to normalize output. A spectrophotometer was used to measure FITC concentration at an excitation wavelength of 485 nm and an emission wavelength of 528 nm.

### Measuring Lengths of Intestinal Segments

Prior to intestinal sample collection, lengths of the small intestine (i.e., proximal segment attached to gizzard to ileo-ceco-colic junction), ceca loops, and colon (i.e., ileo-ceco-colic junction to cloaca opening) were measured via ruler. The average length of the two ceca loops per bird was used as the single value for ceca length per replicate.

### Scanning Electron Microscopy of the Distal Ileum

Roughly 2 cm segments of distal ileum (*n* = 4 per group per time point) were fixed in SEM fixative (4% paraformaldehyde, 3% glutaraldehyde and 0.1 M cacodylate buffer at pH 7.4) at 4°C until proceeding to next step. Tissues were washed with fresh SEM fixative overnight at RT, followed by two 15 min washes in SEM fixative. Then, tissues were post-fixed with 1% osmium tetroxide (0.1 M cacodylate buffer) for 1 h and washed in water for 15 min. For dehydration, tissues were then incubated with 50, 70, 85, and 95% ethanol for 1 h each, followed by overnight incubation with 100% ultrapure ethanol at RT. Then, specimens were dried to critical-point drying via liquid carbon dioxide as the medium and placed in a desiccator until ready to image. Prior to imaging, samples were coated with platinum and examined with a Hitachi S-800 scanning microscopy microscope at 10 kV.

### Analyses of Paraffin-Embedded Ileo-Ceco-Colic Junction

The ileo-ceco-colic junction of each bird were placed into 4% PFA and stored at RT. Subsequently, 5 μm paraffin-embedded cross sections were stained with hematoxylin and eosin (H&E) to assess gut inflammation. More-specifically, parameters measuring inflammation (i.e., focal, multifocal, diffuse), infiltrate (i.e., presence of heterophils, lymphocytes, macrophages as well as hemorrhages), necrosis (i.e., focal, multifocal, diffuse), and location (i.e., lamina propria, villous lamina propria, crypt lamina propria) were used. Furthermore, sections were stained with Alcian blue to enumerate goblet cells. Analyses were performed by a certified pathologist at Iowa State University.

### Bactericidal Assays Against *Salmonella*

To collect small intestinal scrapings (SISs), a 10-cm segment aligning Meckel's diverticulum in the center was longitudinally-cut, excess luminal contents were removed, and the epithelial layer was gently scraped and resuspended with 10 ml phosphate-buffered saline (PBS). Conicals were centrifuged at 5,000 × g for 20 min at 4°C, and 1 ml supernatant was added to 30 μl storage mixture (1% sodium azide, 5% BSA, 50 mM phenylmethane sulfonyl fluoride) before storage at −80°C. Importantly, SISs were confirmed to be Enterobacteriaceae-negative via plating on MacConkey agar. To determine broad protection against *Salmonella*, several *S. enterica* strains (Table 1) were cultured on LB agar (0.1% glucose). Individual colonies were added to PBS until OD_600_ reached 0.1, and this inoculum was diluted until 10^2^ CFU/100 μl was reached. SISs were pooled into two groups per treatment at each time point (A, *n* = 4; B, *n* = 3), and pooled washes were added to *Salmonella* inoculum at 1:1 ratio and incubated for 6 h at 37°C. Solutions were then serially diluted and plated on MacConkey for bacterial enumeration. SIS bactericidal assays were run in triplicate.

**Table 1:** Summary of *Salmonella* isolates tested for *in vitro* resistance assays.

***Salmonella enterica* serovar**	**Isolation source/bank number; relevant antibiotic-resistance profiles and/or characteristics**	**References**
UK-1 (Typhimurium)	Highly-virulent “universal killer,” isolated from horse	(16)
Kentucky	Poultry-isolate; TC_R_, ST_R_, CP_R_, ammonium-resistance	(17)
Albert	Bank number 0401; ST_R_, TC_R_, CP_R_, CA_R_, SU_R_, AG_R_	CDC
Heidelberg	Bank number 0404; CP_R_	CDC
Typhimurium	Bank number 0408; ST_R_, TC_R_, CP_R_, CA_R_, SU_R_	CDC

*TC, tetracycline; ST, streptomycin; CP, cephalosporin; CA, chloramphenicol; SU, sulfanomide; AG, aminoglycoside. Subscript “R” indicates resistance*.

### Small Intestinal Total IgA

Ninety-six-well plates were coated with 0.25 μg/ml unlabeled mouse anti-chicken IgA (i.e., total IgA; H+L, ThermoFisher) overnight at 4°C. CON and SPORE SISs were diluted 1:1 in SEA blocking buffer (ThermoFisher), serially diluted 1:2, and incubated for 1 h at RT. Goat-anti-chicken-IgA-AP (H+L, ThermoFisher) was added, followed by PNPP substrate (ThermoFisher), and absorbance was measured at 405 nm. To measure antibody titer, the reciprocal of the highest dilution values doubling the control value (i.e., CON birds) were considered positive. ELISAs were done in duplicate per individual bird (*n* = 7 per group per time point) and independently-replicated twice.

### Chicken-Specific Immunometabolic Kinome Peptide Array

Distal ileum tissues (*n* = 4 per time point per group) were flash-frozen and stored at −80°C and transported overnight on dry ice to the University of Delaware. For kinome peptide array analyses, ileum tissues from four birds were used per group. Peptide array protocol was carried out as previously described and summarized below (12). Briefly, 40 mg of tissue samples were used for the kinome peptide array protocol. Samples were homogenized by a Bead Ruptor homogenizer in 100 μl of a lysis buffer containing protease inhibitors. Homogenized samples were then mixed with an activation mix containing ATP and applied to peptide arrays. Arrays were incubated in a humidity chamber at 37°C with 5% CO_2_, thus allowing kinases to phosphorylate their target sites. Samples were then washed off the arrays, and a fluorescent phosphostain was applied. Stains not bound to phosphorylated sites were removed by a destaining process. Arrays were then imaged using a Tecan PowerScanner microarray scanner (Tecan Systems, San Jose, CA, USA) at 532–560 nm with a 580 nm filter to detect dye fluorescence. Array images were then gridded using GenePix Pro software (Molecular Devices, San Jose, CA, USA), and the spot intensity signal was collected, thus ensuring peptide spots were correctly associated with their phosphorylation sites. Greater intensity fluorescence correlates to greater phosphorylation at the target site. Fluorescent intensities for treatments were then compared with controls using PIIKA2 (18). The resulting data output was then used in downstream applications such as STRING (19) and KEGG (20) databases used to pinpoint changes in the protein–protein interactions and signal transduction pathways.

### Statistical Analyses

Statistical comparisons for weight gain, intestinal segment length, gut permeability, *Salmonella* killing, and IgA production were performed via Student's *t*-test on GraphPad Prism software. For the kinome array, collected signal intensity values from the scanned array image were arranged into the PIIKA2 input format in Excel. The resultant data were then analyzed by the PIIKA2 peptide array analysis software (http://saphire.usask.ca/saphire/piika/index.html). Using the normalized data set, we performed comparisons between treatment and control groups, calculating fold change (= treatment/control) and a significance *P*-value. The *P*-values were calculated by conducting a one-sided paired *t*-test between treatment and control values for a given peptide. The resultant fold change and significance values were used to generate optional analysis (heatmaps, hierarchical clustering, principal component analysis, pathway analysis) via standard R statistical functions or online analysis platforms.

## Results

### Chloroform-Treated ISs Enhanced Spore Enrichment

**Figure 1:**
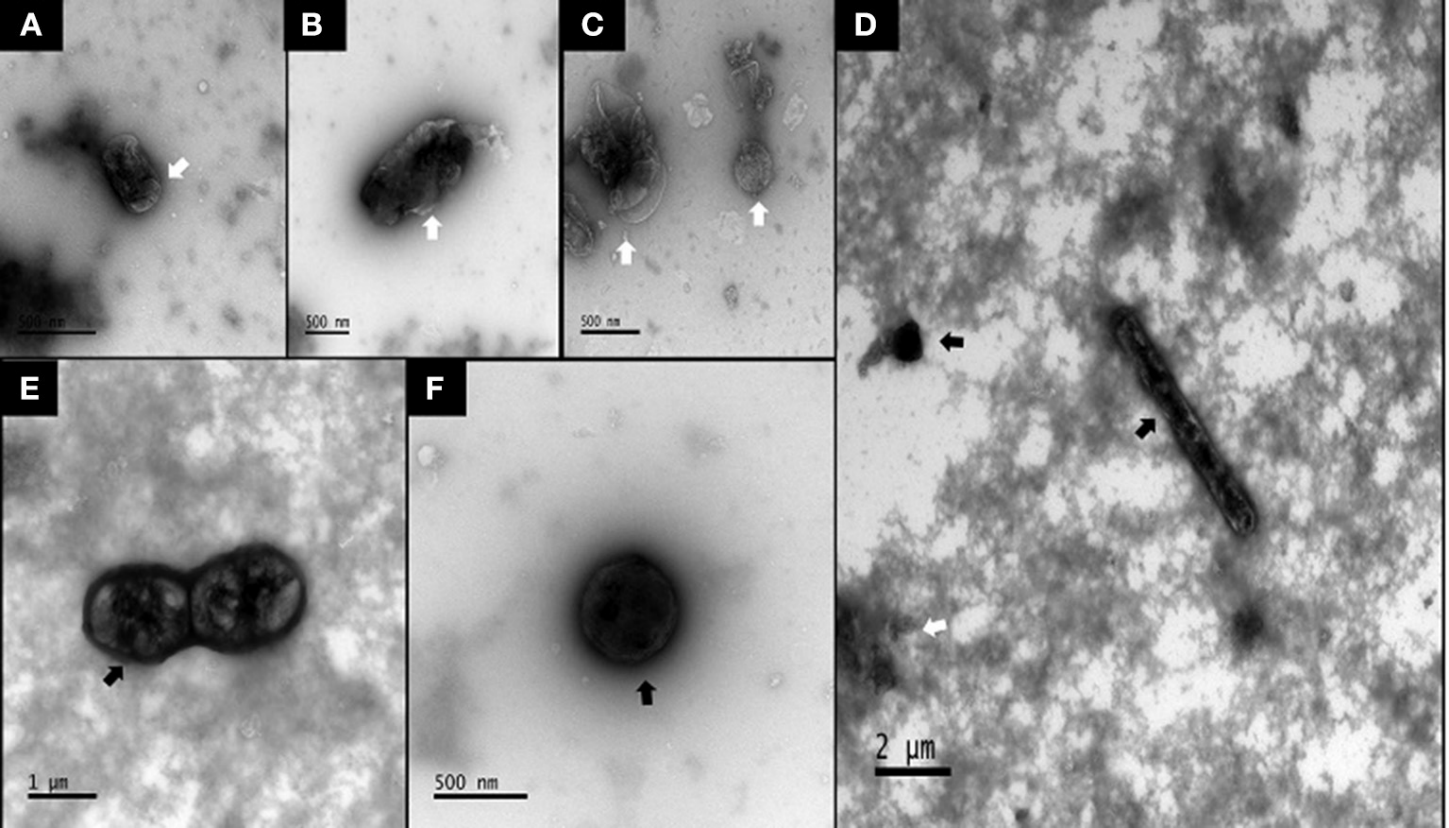
TEM images of bacterial spores in SPORE and CON inocula. **(A,B)**, CON inoculum. **(C–F)**, SPORE inoculum. Atrophied or dead microbes are indicated by faint bodies with contorted outer membranes (white arrows). Spores or viable bacteria are seen as electron-dense (i.e., dark) with discernable membranes (black arrows).

**Figure 2:**
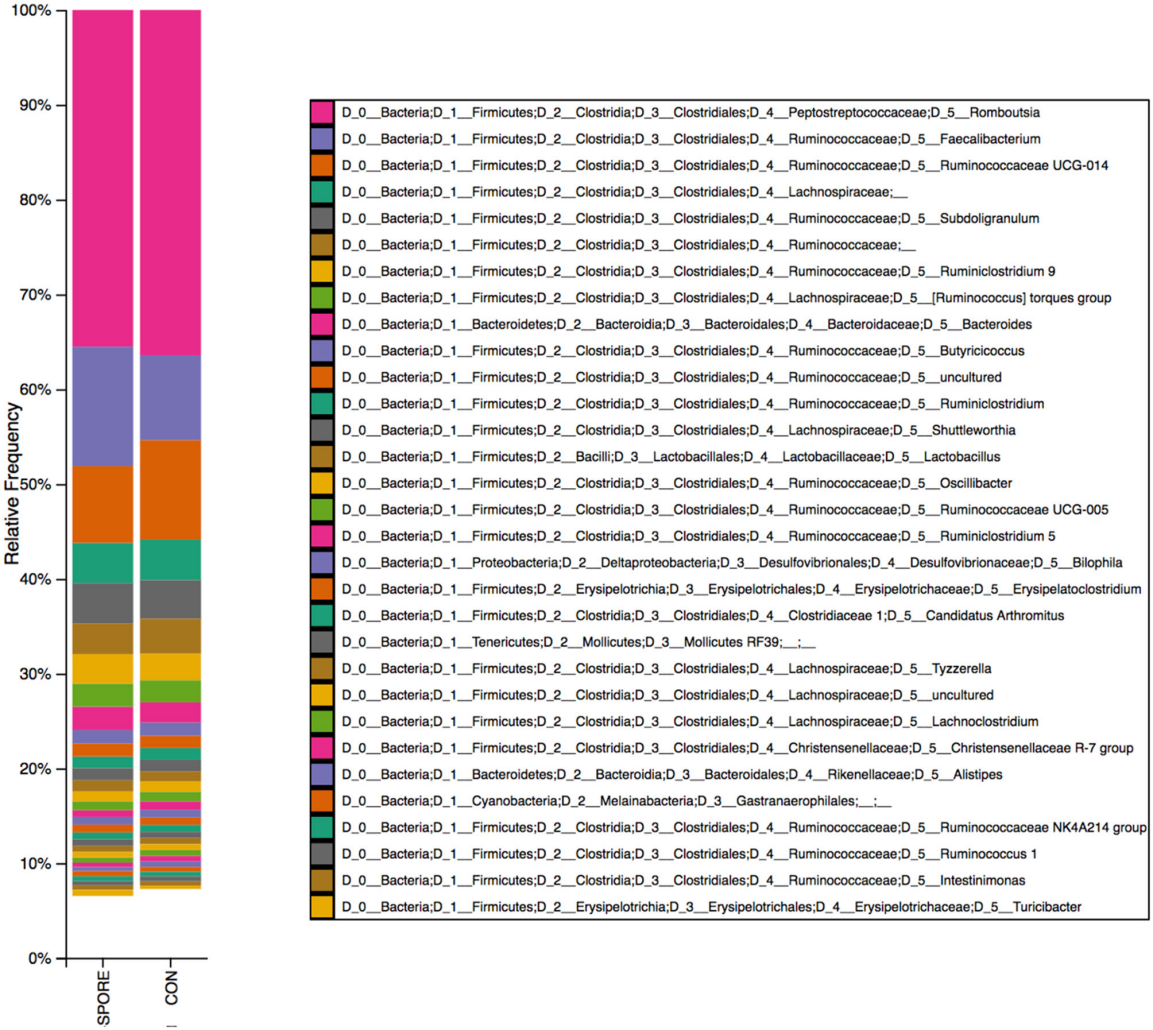
16S reads identified in SPORE and CON inocula. Bacterial taxa from spore-enriched (SPORE) and control (CON) inocula were identified via 16S rRNA gene sequencing and QIIME2 pipeline analysis.

Using transmission electron microscopy (TEM), we did not detect any spores in non-chloroform treated samples, in which bacterial cells exhibited atrophy and death (Figures 1A,B). Although chloroform-treated ISs showed some cellular atrophy (Figure 1C), spores were widely observed (Figures 1D–F) at ~10 spores per μl. Using 16S sequencing, we found that bacterial abundances were highly similar in SPORE and CON inocula, with non-spore (ex: *Faecalibacterium, Subdoligranulum, Butyricoccus*), and spore-forming (ex: *Candidatus* Arthromitus, *Romboutsia*) Clostridia (Figure 2). No *Bacillus* species were detected. The lack of healthy microbes in the CON inoculum suggests the 16S rRNA gene sequences detected in CON inoculum do not indicate viable bacteria but rather dead cells (21, 22).

### SFB Colonize Gut at Much-Earlier Age in SPORE Birds

**Figure 3:**
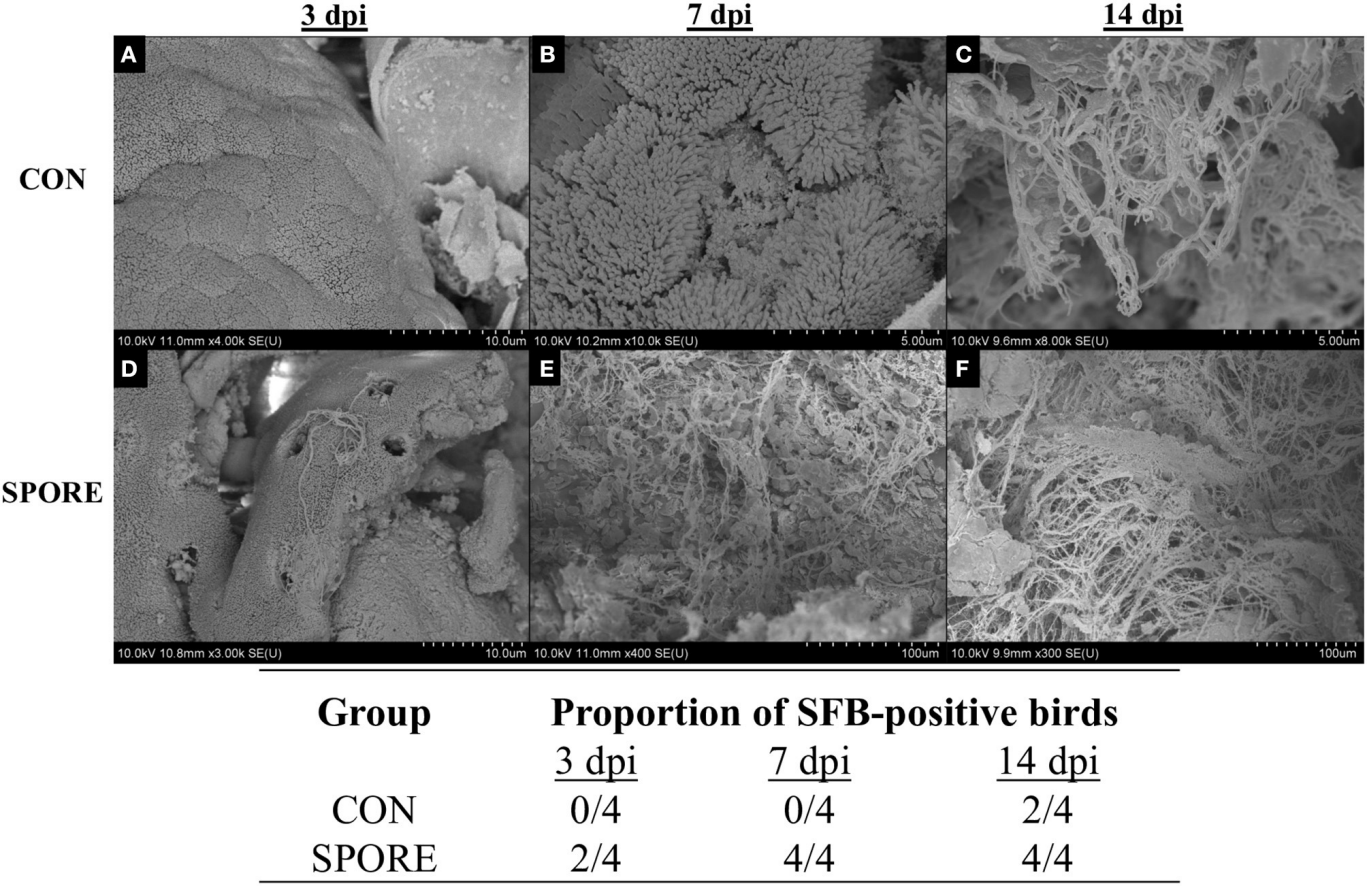
SEM detection of SFB in the distal ileum. SEM images were taken for CON **(A–C)** and SPORE **(D–F)** birds at multiple days post-inoculation (dpi) to track SFB colonization over time. **(A,D)**, 3 dpi. **(B,E)**, 7 dpi. **(C,F)**, 14 dpi. In addition, a table summarizing the total findings is provided.

**Figure 4:**
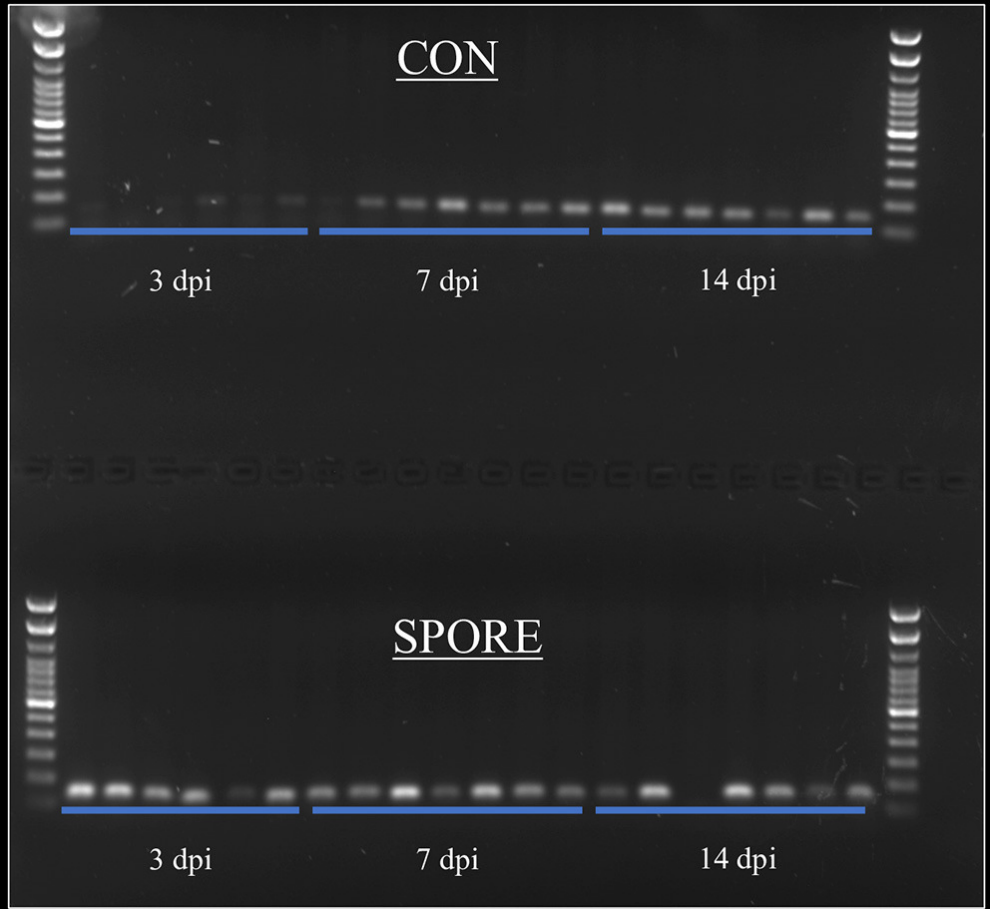
SFB detection in ceca content via PCR. SFB-specific primers were used to detect SFB in ceca content at 3, 7, and 14 dpi time points.

SFB colonization is considered a biomarker for healthy poultry animals (23–25). It plays a major role in immune maturation in mammalian models (8). SFB exclusively-attach to the distal ileum compared to other commensal bacteria [reviewed in (26)] and are routinely-visualized via scanning electron microscopy (SEM) (23, 27). SFB observations from SEM imaging are visually-represented and summarized in Figure 3. In CON group, SFB filaments were absent in chicks at 3 (Figure 3A) and 7 dpi (Figure 3B) and were only detected at 14 dpi in 50% of birds (Figure 3C). However, in the SPORE-treated group, SFB filaments were detected at 3 dpi in 50% of birds (Figure 3D) and in all birds tested at 7 (Figure 3E) and 14 dpi (Figure 3F). Given the murine cecum serves as a reservoir for SFB (8), we investigated the presence of SFB in the chicken ceca content via PCR. SFB were detected in the chicken ceca in all SPORE birds at 3 dpi, whereas SFB was not consistently-detected in the ceca of CON birds at 3 dpi (Figure 4). Nearly all CON and SPORE birds were positive for SFB in the ceca at 7 and 14 dpi (Figure 4).

### SPORE Birds Had Reduced Weight Gain and Gut Permeability

**Figure 5:**
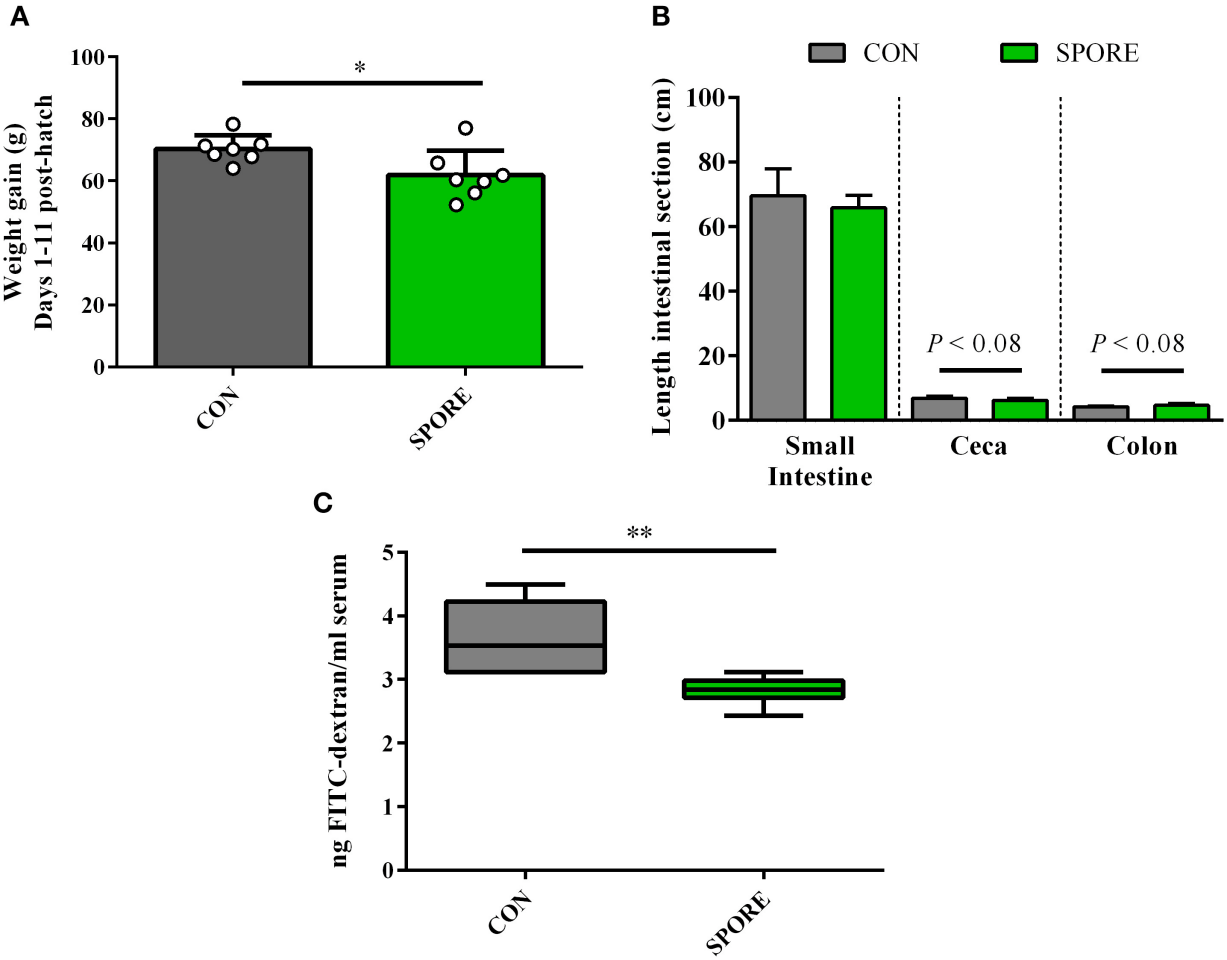
Measures of weight gain and gut morphology. **(A)** Chick weight was measured at 1 and 11 days post-hatch to assess average weight gain per animal. **(B)** Intestinal segment lengths were measured via ruler at 14 days post-inoculation (dpi). **(C)** Gut permeability was measured at 3 dpi via orally-delivered FITC-dextran leakage in serum. ^*^P < 0.05, ^**^P < 0.01.

Tracking weights from 1 to 11 days post-hatch (Figure 5A), SPORE birds had slightly-reduced weight gain (61.89 ± 2.99 g) vs. CON birds (70.27 ± 1.67 g; *P* < 0.05). This finding was independent of feed intake, as net feed consumption during this time frame was nearly-identical between groups (Supplementary Figure 1; CON, 80.7 g; SPORE, 83.2 g). Measuring intestinal segment lengths 14 days post-treatment (Figure 5B), the small intestine length was similar among groups (CON, 69.51 ± 3.18 cm; SPORE, 65.81 ± 1.48 cm). Although not statistically-significant, CON ceca lengths (6.90 ± 0.24 cm) were longer than SPORE lengths (6.24 ± 0.24 cm; *P* < 0.08), and SPORE colon lengths (4.19 ± 0.09 cm) were longer than CON lengths (4.67 ± 0.24 cm; *P* < 0.08). However, at 3 dpi, gut permeability was significantly reduced in SPORE (2.82 ± 0.09 ng FITC dextran/ml serum) vs. CON birds (3.61 ± 0.21 ng FITC dextran/ml serum; Figure 5C; *P* < 0.01), but no differences were seen 7 nor 14 dpi (Supplementary Figure 2). Additionally, treatment did not affect inflammation (hematoxylin and eosin, H&E; Supplementary Figure 3) nor goblet cell enumeration (Alcian blue; data not shown) in the ileo-ceco-colic junction of birds at any time point.

### SPORE SISs *in vitro Salmonella* Killing Was Time-Dependent and IgA Independent

**Figure 6:**
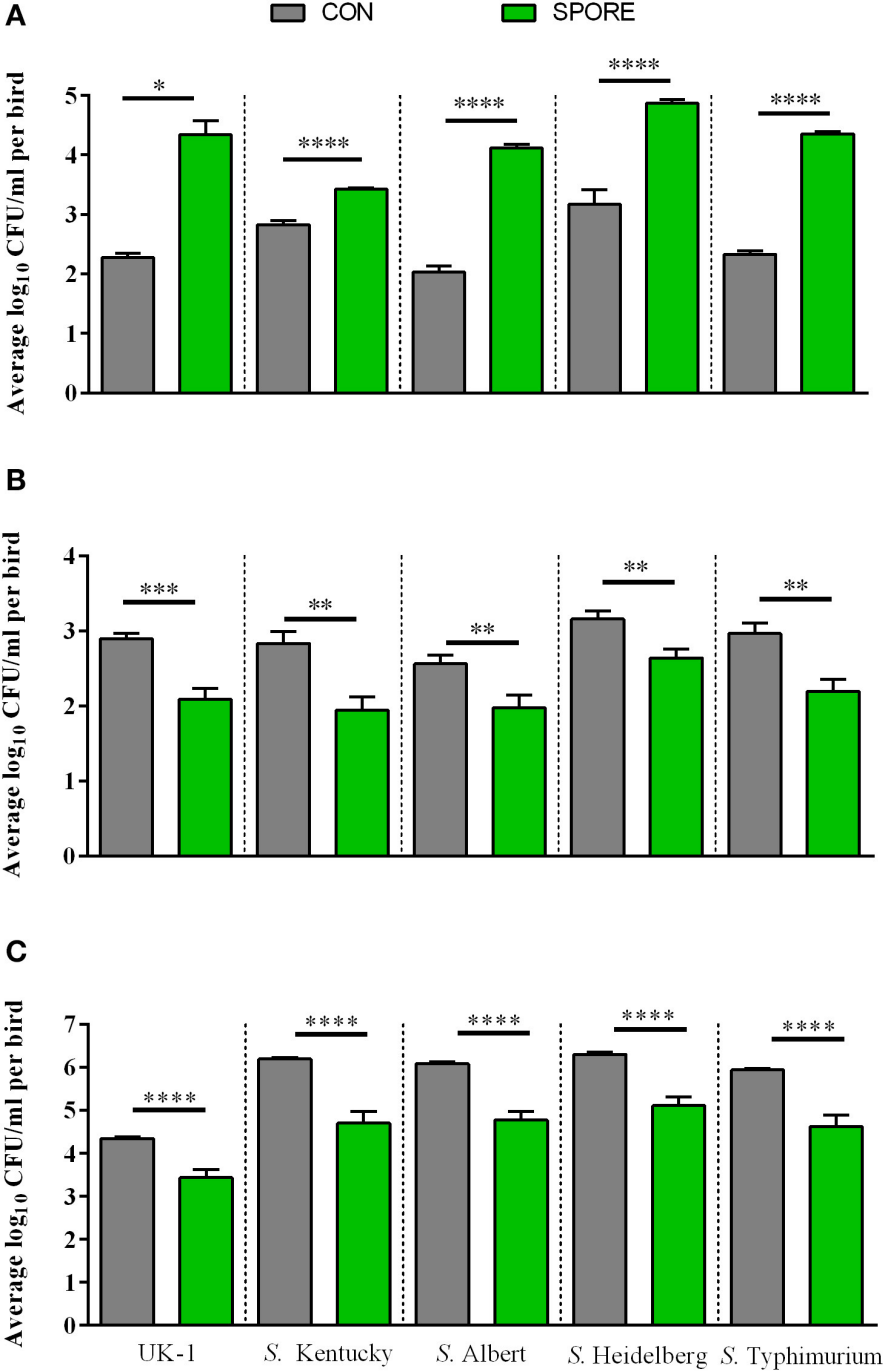
*Salmonella* resistance assays *in vitro*. *Salmonella enterica* resistance was measured using *in vitro* bactericidal assays against multiple *Salmonella* isolates (summarized in Table 1). *Salmonella* killing was performed in small intestinal scrapings taken at 3 **(A)**, 7 **(B)**, and 14 dpi **(C)** in experimental duplicate. ^*^P < 0.05, ^**^P < 0.01, ^***^P < 0.001, and ^****^P < 0.0001.

**Figure 7:**
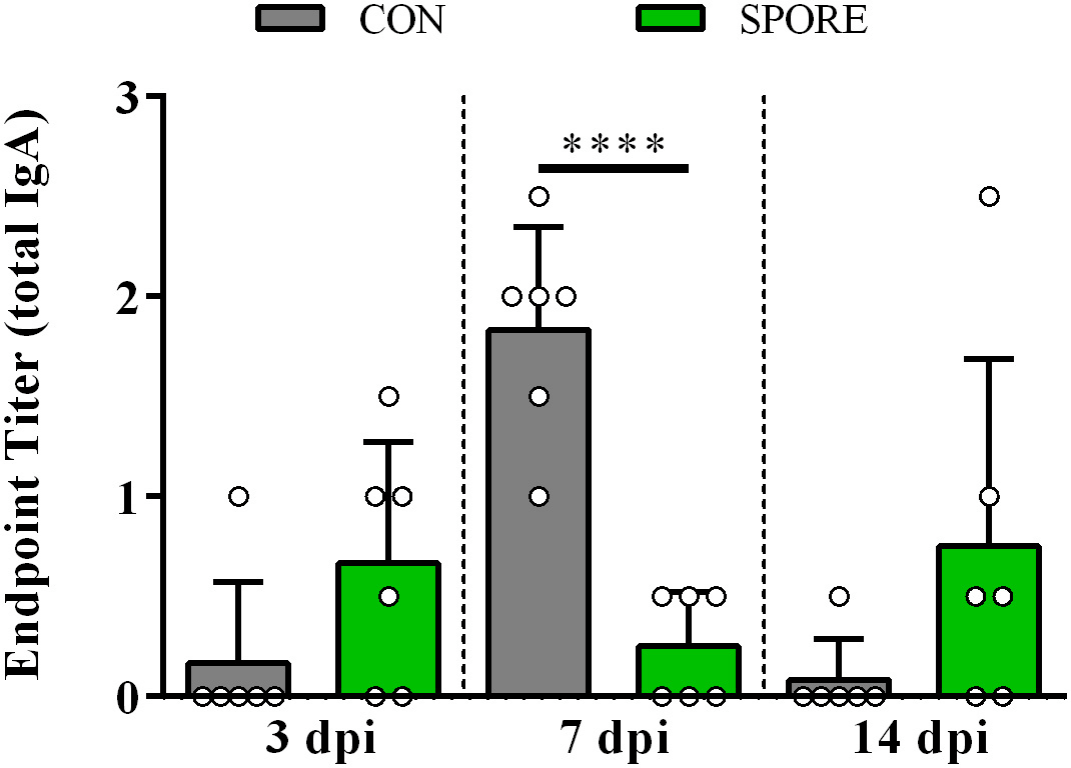
Total IgA production. Endpoint titers for total IgA were measured in small intestinal scrapings from birds at 3, 7, and 14 days post-inoculation (dpi) via ELISA. Assays were performed in experimental duplicate. ^****^P < 0.0001.

Using small-intestinal scrapings (SISs) for *in vitro Salmonella*-killing assays, we found that SPORE SISs at 3 dpi (Figure 6A) were highly reduced in inhibiting the growth of every *S. enterica* serovar tested vs. CON (*P* < 0.05). However, at 7 (Figure 6B) and 14 (Figure 6C) dpi, SPORE SISs had greater growth-suppression of all *S. enterica* serovars tested vs. CON (*P* < 0.05). Measuring total IgA in SISs at each time point (Figure 7), endpoint titers were similar at 3 and 14 dpi between SPORE and CON birds. However, total IgA levels were significantly lower at 7 dpi in SPORE compared to CON birds (*P* < 0.0001).

### Several Immune and Metabolic Pathways Were Globally Altered in SPORE Birds

Chicken-specific kinome arrays were used to assess changes to ileal immunometabolism (12). Kinome analysis was carried out on the ileal samples taken from SPORE and CON chickens at 3 and 7 dpi (*n* = 4 per group per time point). The results from the biological replicates from each group (SPORE and CON) and time point were combined to provide a representative result. To remove any non-specific or baseline phosphorylation signals from the analysis, data from each time point was compared to matched controls. Kinome data were subjected to pathway overrepresentation analysis to determine which cellular pathways/processes are activated under the SPORE conditions as compared to time-matched CON. To ensure that the identified pathways represent conserved and consistent biological responses, input data were limited to peptides with a consistent pattern of differential phosphorylation across the four biological replicates as well as significant changes (*P* ≤ 0.05) in phosphorylation level relative to CON. These peptides for each time-point were input into the Search Tool for the Retrieval of Interacting Genes/Proteins (STRING) database (19). Using STRING functionality, Kyoto Encyclopedia of Genes and Genomes (KEGG) (20) pathway results were generated for each dataset. The STRING-generated KEGG-pathway results showed several pathways altered by spore inoculation at a statistically significant level [*P* ≤ 0.05 false discovery rate (FDR) corrected].

Several immune and metabolic signaling pathways were dramatically altered by SFB treatment. The top 19 immunologic and 18 metabolic altered pathways are shown in Tables 2, 3, respectively. Both immunologic and metabolic KEGG pathways had multiple peptide phosphorylation events altered at both time-points post-inoculation. In total, 414 differentially phosphorylated peptides from immune pathways were observed in the ileum of chickens 3 dpi and 389 differentially phosphorylated immune peptides on 7 dpi (Table 2), signifying a dramatic local post-translational modification of the immune proteins induced by the SPORE treatment. Of the 414 immune-related peptides differentially phosphorylated at 3 dpi, 109 belong to the innate immune signaling pathways including pattern recognition receptor signaling (TLR and NOD), NK cell signaling, and Fc receptor phagocytosis (ε and γ) while 95 immune-related peptides belonging to the acquired immune signaling pathways including the T cell receptor signaling, IL17 signaling, and JAK-STAT pathways. By 7 dpi, there was a reduction in SPORE innate immune signaling compared to SPORE birds at 3 dpi: 81 total peptides from the TLR and NOD signaling, NK cell signaling and Fc receptor phagocytosis signaling. Conversely, by 7 dpi there was an increase in the number of phosphorylated peptides in SPORE from the acquired T cell signaling pathways: 109 including T cell receptor signaling, T_H_17 differential signaling, IL17 signaling, and JAK-STAT pathways compared to SPORE birds at 3 dpi. More specifically, T_H_17 signaling was only increased in SPORE birds compared to CON at 7 dpi (Table 2).

**Table 2:** KEGG immune pathways enriched from unique peptides in SPORE (compared to CON) birds 3 and 7 days post-inoculation.

**KEGG pathway**	**3 dpi**		**7 dpi**	
	**# Proteins**	***p-*value (FDR)**	**# Proteins**	***p-*value (FDR)**
PI3K-Akt signaling	56	7.73 10^−33^	45	1.75 × 10^−27^
Chemokine signaling	34	3.71 × 10^−22^	29	4.11 × 10^−20^
B cell receptor signaling	25	6.10 × 10^−22^	21	2.39 × 10^−19^
T cell receptor signaling	26	2.70 × 10^−20^	25	1.89 × 10^−21^
Autophagy	28	3.03 × 10^−20^	23	2.01 × 10^−17^
Fc epsilon RI signaling	22	4.95 × 10^−19^	20	1.21 × 10^−18^
Toll-like receptor signaling	25	5.62 × 10^−19^	18	1.15 × 10^−13^
Fc-gamma R-mediated phagocytosis	22	6.25 × 10^−17^	18	1.56 × 10^−14^
Natural killer cell mediated cytotoxicity	24	2.46 × 10^−16^	19	2.05 × 10^−13^
Apoptosis	23	1.09 × 10^−14^	20	8.23 × 10^−13^
TNF signaling	21	1.74 × 10^−14^	22	1.70 × 10^−17^
JAK-STAT signaling	24	3.12 × 10^−14^	21	1.51 × 10^−13^
IL-17 signaling	18	1.28 × 10^−12^	12	3.62 × 10^−8^
Leukocyte transendothelial migration	19	2.61 × 10^−12^	15	4.26 × 10^−10^
Inflammation mediator regulation of TRP channels	16	1.13 × 10^−10^	14	3.92 × 10^−10^
T_H_17 cell differentiation		NS	15	1.36 × 10^−10^
NF-κB signaling	15	1.13 × 10^−9^	15	4.30 × 10^−10^
Wnt signaling	17	4.96 × 10^−9^	13	4.14 × 10^−7^
NOD-like receptor signaling	17	3.69 × 10^−8^	12	1.04 × 10^−5^

*Proteins statistically significantly differentially phosphorylated uniquely in the distal ileum from the SPORE group were pulled out of the array data and input into STRING for analysis. The top 19 immune pathways from each point are shown in this table. FDR, false-discovery rate*.

**Table 3:** KEGG metabolic pathways enriched from unique peptides in SPORE (compared to CON) birds 3 and 7 days post-inoculation.

**KEGG pathway**	**3 dpi**		**7 dpi**	
	**# Proteins**	***p-*value (FDR)**	**# Proteins**	***p-*value (FDR)**
Insulin signaling	39	5.43 × 10^−31^	29	3.72 × 10^−23^
HIF-1 signaling	31	7.33 × 10^−26^	27	6.42 × 10^−24^
AMPK signaling	30	1.42 × 10^−22^	21	1.22 × 10^−15^
Insulin resistance	28	1.25 × 10^−21^	23	1.21 × 10^−18^
mTOR signaling	31	1.78 × 10^−21^	25	4.25 × 10^−18^
Glucagon signaling	22	4.78 × 10^−16^	16	1.04 × 10^−11^
cAMP signaling	23	9.38 × 10^−12^	20	3.30 × 10^−11^
Glycolysis/gluconeogenesis	14	2.66 × 10^−10^	10	2.00 × 10^−7^
cGMP-PKG signaling	18	3.81 × 10^−9^	15	3.52 × 10^−8^
Calcium signaling	17	1.01 × 10^−7^	10	0.00041
PPAR signaling	11	3.41 × 10^−7^	9	2.88 × 10^−6^
Propanoate metabolism	8	6.58 × 10^−7^		NS
Starch and sucrose metabolism	8	7.88 × 10^−7^	4	0.0023
Fatty acid degradation	8	4.91 × 10^−6^	6	9.98 × 10^−5^
Galactose metabolism	7	6.00 × 10^−6^	4	0.0019
Fructose and mannose metabolism	7	8.54 × 10^−6^	4	0.0023
Carbohydrate absorption and digestion	7	3.30 × 10^−5^	7	7.81 × 10^−6^
Biosynthesis of amino acids	9	1.79 × 10^−5^	7	0.00016

*Proteins statistically significantly differentially phosphorylated uniquely in the distal ileum from the SPORE group were pulled out of the array data and input into STRING for analysis. The top 18 metabolic pathways from each point are shown in this table. FDR, false-discovery rate*.

Concurrently with several changes in peptide-phosphorylation in the ileal immune response, dramatic alterations in the metabolic phenotype were also occurring in the SPORE-treated chickens (Table 3). A total of 389 metabolically associated peptides were altered in the ileum of SPORE-treated chickens compared to CON birds at 3 dpi. Additionally, 101 of these peptides were associated with the mammalian target of rapamycin (mTOR), hypoxia-inducible factor-1α (HIF-1α), and insulin signaling pathways. The mTOR pathway phosphorylation triggers the local tissue's phenotype to anabolic metabolism by increasing the expression of the transcription factor HIF- 1α (28–31). An increase in HIF-1α expression results in the subsequent increased transcription of both glycolytic genes and pro-inflammatory genes (29, 30). Further, there are 30 altered peptides in the 5'-adenosine monophosphate-activated protein kinase (AMPK) pathway at 3 dpi. AMPK is a sensor of cellular metabolism that directly mediates the function of mTOR and switches metabolic phenotype to catabolic metabolism when it senses a change in the AMP: ATP ratio (32, 33). Both alpha and beta subunits of AMPK showed significantly decreased phosphorylation (data not shown), implying that the AMPK pathway was deactivated. Similar results were observed at 7 dpi in the SPORE-treated chickens with 81 altered peptides associated with the insulin, mTOR, and HIF-1α signaling pathways with only 21 peptides in the AMPK pathway.

**Figure 8:**
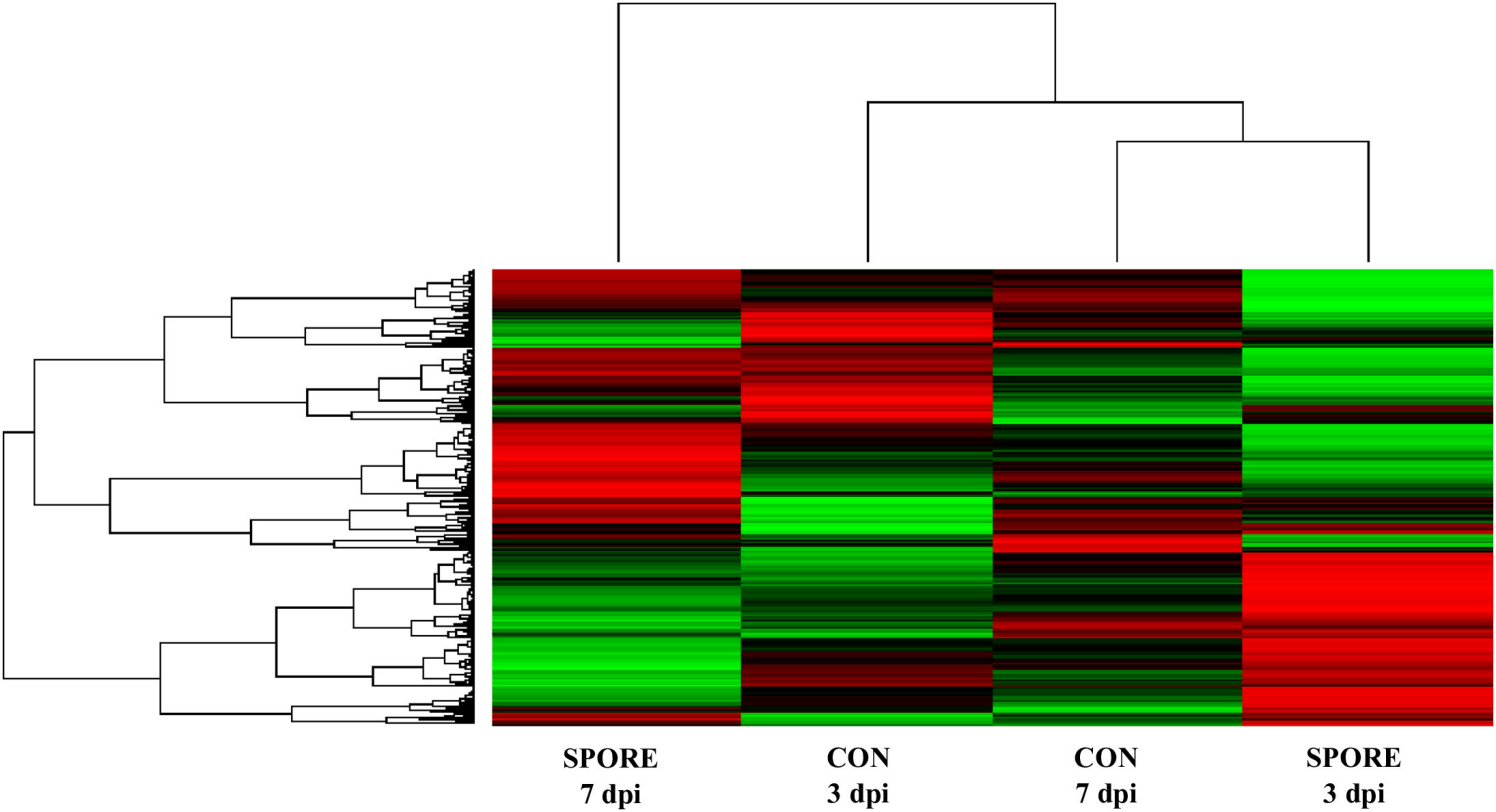
Heatmap and clustering of kinome profiles. The raw kinome signal from the peptide array was input into the custom software package PIIKA 2. Red indicates relative increased phosphorylation, whereas green indicates relative decreased phosphorylation of each peptide on the array.

To determine similarities in kinome profiles (i.e., kinotypes) between treatment groups and time points, Platform for Intelligent, Integrated Kinome Analysis, version 2 (PIIKA2) was used to combine the biological replicates for each treatment and tissue, normalize the data, and generate a representative kinotype that provides a visual image of the differences in phosphorylation events between SPORE and CON. Figure 8 shows that the most similar distal ileum kinotypes were CON from 7 dpi and SPORE from 3 dpi. However, the kinotype of SPORE birds from 7 dpi was the most unique, separating from the other three kinotypes.

## Discussion

Clostridia consists of several spore-forming bacteria that play a major role in the maturation of the gut immune system in mammals. SFB, otherwise known as *Candidatus* Arthromitus or Savagella (34), are widely distributed among animals and were detected in each inoculum via bacterial 16S rRNA gene sequencing. In mice, SFB directly attach to the epithelium without damaging the gut barrier nor causing excessive inflammation (35) and improve epithelial barrier functions (13) as well as resistance to enteric infections like *Citrobacter* and *Salmonella* (8, 36). The limited work on poultry SFB demonstrate they colonize the distal ileum, cecal tonsil, and loops (37) and are associated with improved IgA production (24) and growth performance (25). Other notable Clostridia detected in the inocula include *Faecalibacterium, Butyricicoccus*, and *Subdoligranulum*. *Faecalibacterium prausnitzii* is a butyrate producer that improves anti-inflammatory functions and has been posited as a probiotic candidate in humans [(38); reviewed in (39)]. *Butyricoccus pullicaecorum* was previously isolated from the chicken ceca (40) and demonstrated to have probiotic potential as a butyrate and acetate producer, depending on *in vitro* conditioning (41). *Subdoligranulum variabile*, another butyrate producer, is phylogenetically-similar to *F. prausnitzii* but otherwise has not been well-characterized (42). Importantly, all of these bacterial species are unable to form spores (40, 42, 43), which suggests that the 16S rRNA gene reads for several taxa detected in these inocula were derived from remnants of the cellular fraction of intestinal scrapings and are not indicative of viable bacteria. Since spores were only-observed in the chloroform-treated SPORE inoculum, this suggests that only spore-forming bacteria like SFB (13), *Romboutsia* (44), Lachnospiraceae and Ruminococcaceae (45) would be viable in the SPORE inoculum. Future studies will use fluorescence *in situ* hybridization to assign species-identity to the spores and use live-dead fluorescence sorting to remove dead cells prior to 16S rRNA gene sequencing (21).

Interestingly, although SFB only constituted a minority of the 16S reads, there were vast improvements in their intestinal colonization when delivered via single SPORE inoculum. Thus, we demonstrate that oral inoculation with ileal spores (1) hastens SFB colonization and (2) improves the consistency of SFB colonization between birds. However, it is unclear whether this colonization is facilitated by SFB in the inoculum directly, whether other spore-formers present in the inoculum produce certain metabolites, which improve SFB colonization, or a combination of both mechanisms. A single-dose *in ovo* inoculation of lactic acid-producing bacteria (LAB) increased 16S reads of SFB in the distal ileal microbiome by day 10 post-hatch (46), suggesting that early exposure to LAB or ileal spores promote early colonization of SFB. However, although our study used oral inoculation at day-of-hatch to deliver these ileal spores, comparing this method with *in ovo* delivery would be worth pursuing. Additionally, one of the more notable findings of our study was the broad changes of numerous immunometabolic pathways in SPORE birds, associated with SFB adherence to the distal ileum. This study showed a consistent trend in which phosphorylation shifts in immunometabolic pathways, mainly innate immunity, were reduced from 3 to 7 dpi. Similarly, single-dose *in ovo* delivery of *Citrobacter* species or LAB also altered early inflammatory responses in the chicken gut (47, 48). These findings suggest that innate immune responses are highly responsive to microbial “pioneers” in the chicken intestine. Furthermore, our study specifically-finds that innate immune responses in the ceca peaked early post-inoculation of ileal spores but decreased over time.

As seen in this study, we identified increased phosphorylation of several enzymes within mTOR, insulin, and PI3K/Akt signaling pathways at both 3 and 7 dpi in SPORE birds. The protein mTOR is a serine/threonine, PI3K-related kinase that directs cell metabolism via sensing environmental cues, such as when immune cells are in metabolically-demanding situations during stimulation with immune regulatory signals (32). Additionally, mTOR is incorporated into two protein complexes, mTOR1 and mTOR2. These complexes are essential in regulating nutrient and endocrine signals (mTOR1) as well as proliferation and survival [reviewed in (49)]. The most important role for mTOR2 is the activation of Akt, the key effector in insulin/PI3K signaling (50). In fact, insulin and mTOR signaling pathways are highly coupled and display significant overlap, so much that it is referred to as the insulin/mTOR signaling pathway (51, 52). All of these pathways have been previously reported to interact with the gut microbiota (35, 53–55), suggesting the microbes in the SPORE inoculum are driving these responses. Work is currently underway to more-closely analyze these phosphorylation networks to identify a mechanism in which the ileal spore-inoculum induced kinomic changes over time.

In the current study, SPORE birds exhibited less weight gain than CON birds from 1 to 11 days post-hatch. Given that gut microbes calibrate the chicken gut immune system in early life (56), it is likely the large, immunometabolic shifts seen in this study shifted resources from weight gain to the immune system. Animals that undergo excessive innate and inflammatory responses grow more slowly due to reduced feed conversion efficiency (57). Although weight gain is one of the most important parameters for broiler productivity, it needs to be emphasized that (1) this study investigated the effects in layers, which are immunologically and metabolically-distinct from broilers (58–60) and (2) only the first eleven days post-inoculation were evaluated, confounding conclusions on how this ileal spore treatment could affect broiler productivity. Additionally, several innate immune pathways like Toll-like receptor and JAK-STAT were activated in SPORE birds, suggesting the reduced weight gain is a result of host innate responses to the SPORE inoculum. Although SPORE inoculum activated innate immune pathways, there were no differences in ceca inflammation between SPORE and CON birds based on H&E staining. Thus, this innate inflammation appears to be non-pathological. Although inflammation in the ileum (the primary site of SFB adherence) was not measured, SFB adherence to this site, has not been implicated in ileal inflammation (61). Additionally, alterations in the PI3K/Akt signaling pathway, observed in this study, may also contribute to differences in growth rate between treatment groups (62). Altogether, we speculate that immune-cell populations in the SPORE gut may have utilized metabolic resources that might have otherwise contributed to growth.

SPORE birds exhibited lower gut permeability vs. CON birds, suggesting these ileal spores reduce intestinal leakage. Similarly, the distal ileum of SPORE birds displayed notable changes in Wnt signaling and AMPK signaling. The Wnt pathway is conserved in animals and is crucial for maintaining homeostasis in the gut via differentiation of intestinal stem cells, which then differentiate into several cell-types like enterocytes and Paneth cells [reviewed in (63)]. Furthermore, AMPK signaling is crucial for cell-commitment to enterocytes, and loss of AMPK led to increased barrier leakiness (64). Thus, increased rates of cell differentiation into enterocytes may reduce gut permeability in SPORE birds. Additionally, Feng et al. found that higher levels of Akt phosphorylation and PI3K activation enhance gut barrier integrity (65), suggesting SPORE-induced changes of the PI3K/Akt signaling pathway may also contribute to this reduction in gut permeability. Although we did not look at changes in tight junction protein formation and gene expression in this study, ileal spore treatment may have affected these parameters, as *in ovo* exposure to bacteria induced changes in tight junction signaling (47). Future studies will evaluate expression of tight junction proteins to allow better-interpretation of gut permeability data.

Paneth and plasma cells in the small intestine primarily release immunological effectors like host-defense peptides (HDPs) and IgA, respectively, making analyses of SISs an effective means of conveniently assessing gut immunity *in vitro*. These factors are crucial for regulating the gut microbiota (66, 67), including bacterial pathogens like *Salmonella*. In this study, SISs from SPORE birds at 7 and 14 dpi exhibited superior bactericidal activities against all *Salmonella* isolates vs. CON, albeit resistance was reduced at 3 dpi. However, the observed resistance at later time points suggests killing increases over time, although this remains to be explored *in vivo*. *In vivo* reduction of *Salmonella* Enteritidis in cecal tonsils and content was observed in birds given FloraMax®-B11 *in ovo* (68), suggesting that a single, early exposure to certain bacteria promote anti-*Salmonella* responses. These patterns in *Salmonella* resistance are notable, as poultry products are a major source of salmonellosis in the United States (69). However, these assays are not entirely-reflective of *in vivo* conditions, as cell-mediated mechanisms like heterophil infiltration (70) as well as microbiota-dependent mechanisms like competitive exclusion (71). Rather, this assay is a basic screen to identify differences in bactericidal or bacteriostatic effector molecules present in SISs. *In vivo* tests against *Salmonella* will be explored in future studies.

*Salmonella* resistance was independent of IgA levels, as total IgA levels were not positively associated with bactericidal responses. Thus, antimicrobial products like HDPs were likely driving these responses. Uniquely, SPORE birds had reduced *Salmonella in vitro* resistance at 3 dpi, aligning with the absence of T_H_17 cell differentiation pathway-activation in the distal ileum. Innate production of HDPs like gallinacins is elevated in the intestine of chicks at post-hatch for the first three days of life and drops after day four (72), which explains why CON birds exhibited greater *Salmonella* killing at 3 dpi independent of T_H_17 signaling. Similar to SFB-colonized mice (8), we report phosphorylation of the T_H_17 cell differentiation pathway in SPORE chickens at 7 dpi alone, a shift from T_H_1 and T_H_2 differentiation observed at 3 dpi. Supporting this hypothesis, propionate metabolism was similarly-phosphorylated at only 3 dpi in SPORE birds. Propionate has been demonstrated *in vitro* to increase T_H_17 differentiation via promoting phosphorylation of an mTOR target protein (73). Under normal conditions, T_H_17 responses emerge around 16 days post-hatch (72), suggesting this treatment hastens maturation of the gut immune defenses. Thus, the elevated resistance to *Salmonella* in SPORE SISs at 7 and 14 dpi may result via a propionate-dependent, T_H_17 cell-mediated increase in HDP production.

One notable observation was that total IgA production was markedly lower at 7 dpi in SPORE birds compared to controls. Similarly, as mentioned earlier, early HDP-production at 3 dpi appears downregulated by SPORE treatment (as indicated by reduced *Salmonella* resistance vs. CON birds). In chickens, SFB abundances naturally peak between 2 and 3 weeks post-hatch. After this point, SFB levels fall as IgA levels begin to rise in the chicken intestine (24). This demonstrates an aversion to host-derived IgA by SFB, supported in studies in which IgA*-*deficient mice exhibit increased SFB colonization (15, 74). Thus, given SFB and other microbes in the SPORE inoculum may be susceptible to humoral immune effectors like HDPs and IgA, we hypothesize these ileal spores elicit signals to downregulate innate-HDP and total IgA production in early life to facilitate initial colonization in the chicken intestine. However, HDP production appears to drastically-increase by 7 dpi in SPORE birds, likely due to changes in adaptive immunity (i.e., T_H_17 signaling) rather than innate mechanisms.

### Conclusion

This study demonstrated for the first time that SFB spore-containing inoculum reduces gut permeability in young chickens and stimulates innate and adaptive immune responses. Additionally, we are the first to study immunometabolic signaling induced by host-SFB interactions at the kinomic level. SFB-based treatment can potentially-protect chickens from enteric pathogens, partly due to its unique ability to trigger a T_H_17 response, which provides evidence of its potential as an agricultural treatment for poultry animals. Current efforts are underway to monoculture SFB *in vitro* to study its direct impacts on the chicken gastrointestinal tract as well as determine if common storage practices (i.e., lyophilization) can improve long-term maintenance of these intestinal spores.

## Data Availability Statement

The datasets presented in this article are not readily available because Data were generated and are maintained by MK. Requests to access the datasets should be directed to mike.kogut@usda.gov.

## Ethics Statement

The animal study was reviewed and approved by 18-386 and 19-072.

## Author Contributions

GR and MM conceived, designed the experiments, and wrote the manuscript. GR, RA, MK, and MM performed the experiments, analyzed the data, and revised the manuscript. RA, MK, and MM contributed reagents, materials, and analysis tools. All authors read and approved the final manuscript.

## Conflict of Interest

The authors declare that the research was conducted in the absence of any commercial or financial relationships that could be construed as a potential conflict of interest.
